# Reactive Oxygen Species and NOX Enzymes Are Emerging as Key Players in Cutaneous Wound Repair

**DOI:** 10.3390/ijms18102149

**Published:** 2017-10-15

**Authors:** Dominik André-Lévigne, Ali Modarressi, Michael S. Pepper, Brigitte Pittet-Cuénod

**Affiliations:** 1 Department of Plastic, Reconstructive & Aesthetic Surgery, University Hospitals of Geneva, 1205 Geneva, Switzerland; ali.modarressi@hcuge.ch (A.M.); brigitte.pittet-cuenod@hcuge.ch (B.P.-C.); 2 Department of Human Genetics and Development, Faculty of Medicine, University of Geneva, 1206 Geneva, Switzerland; michael.pepper@up.ac.za; 3 SAMRC Extramural Unit for Stem Cell Research and Therapy, and Institute for Cellular and Molecular Medicine, Department of Immunology, Faculty of Health Sciences, University of Pretoria, Pretoria 0002, South Africa

**Keywords:** reactive oxygen species, NADPH oxidase, NOX enzymes, wound repair, wound healing, chronic wounds, wound contraction, re-epithelialization, hyperbaric oxygen therapy, oxidative stress

## Abstract

Our understanding of the role of oxygen in cell physiology has evolved from its long-recognized importance as an essential factor in oxidative metabolism to its recognition as an important player in cell signaling. With regard to the latter, oxygen is needed for the generation of reactive oxygen species (ROS), which regulate a number of different cellular functions including differentiation, proliferation, apoptosis, migration, and contraction. Data specifically concerning the role of ROS-dependent signaling in cutaneous wound repair are very limited, especially regarding wound contraction. In this review we provide an overview of the current literature on the role of molecular and reactive oxygen in the physiology of wound repair as well as in the pathophysiology and therapy of chronic wounds, especially under ischemic and hyperglycemic conditions.

## 1. Introduction

Oxygen is needed in virtually every step of wound repair as the energy needed during biosynthesis, intracellular transportation and cell movement relies on adenosine triphosphate (ATP), which is most efficiently synthesized in an oxygen-dependent manner. During wound repair, high proliferation rates and the production of extracellular matrix (ECM) components (most notably collagen) increase the demand for energy and thus for oxygen [1].

Oxygen supply at the wound site is dependent on a variety of different factors including pulmonary gas exchange, cardiac output, peripheral perfusion rate, capillary density in the wound and its surroundings, and the oxygen consumption rate of reparative cells in the wound [2]. Oxygen delivery from the capillaries to the cells relies exclusively on diffusion and is therefore dependent on arterial oxygen tension and perfusion [3]. The distance between cells in the granulation tissue and the next capillary is of crucial importance [4,5].

Wound repair is dependent on oxygen not only for energy supply but also because it is needed in a variety of key enzymatic reactions. In fact, over the last few decades the understanding of the role of oxygen in cell physiology has evolved from its long-recognized importance as an essential factor in oxidative metabolism and its germicide role in host defense to its recognition as an important player in cell signaling [6,7].

Reactive oxygen species (ROS) are small oxygen-derived molecules that are either oxidizing agents or are easily converted into oxygen radicals. They react with a variety of molecules including other small inorganic molecules, carbohydrates, lipids, proteins, and nucleic acids [8]. It is widely accepted that ROS are major contributors to cell damage during the ageing process [9]. However, ROS also play beneficial roles. The concerted production of large amounts of ROS by immune cells is of great importance for effective host defense [10]. In addition, it has become increasingly clear that ROS (in low concentrations) are involved in a myriad of physiological cell signaling pathways, referred to as redox signaling pathways [8]. There is also increasing evidence that ROS are crucial for wound repair, not only as germicides but also for cellular signaling [11,12].

ROS are produced physiologically as byproducts of other biological reactions involving mitochondria, peroxisomes, cytochrome P-450, and many others [13]. However, ROS can also be produced specifically by specialized enzymes, notably nicotinamide adenine dinucleotide phosphate (NADPH) oxidases of the NOX family. NOX enzymes are membrane-bound complexes that transport electrons across biological membranes to reduce oxygen to superoxide (O2-) [8]. Superoxide then reacts to form other reactive oxygen species, such as hydrogen peroxide (H_2_O_2_), peroxide anion (HO_2_^−^), and hydroxyl radical (^•^OH) [14], which are involved in a number of different cellular functions including differentiation, proliferation, apoptosis, migration, and contraction [8,11,15]. Seven isoforms of NOX enzymes have been described: NOX1, NOX2 (classic phagocyte oxidase), NOX3, NOX4, NOX5, DUOX1, and DUOX2. Despite their similar structure, they differ in their function and mechanism of activation [8]. Oxygen being their principal substrate, all members of the NOX family are strictly depending on oxygen and it has been reported that they require in vitro oxygen tensions between 40 and 80 mmHg to work at 50% enzymatic speed [14,16].

Wound repair is a complex, dynamic, and interactive process involving a variety of cells, soluble mediators, and extracellular matrix components, implicated in the processes of coagulation, inflammation, angiogenesis, re-epithelialization, fibroplasia, and contraction. Wound repair is traditionally described in four highly interconnected phases: (1) coagulation, (2) inflammation, (3) proliferation, and (4) maturation [17]. The two main mechanisms leading to wound closure during the proliferation phase are re-epithelialization and contraction, the latter leading to a reduction in wound size more rapidly than the former. NOX isoforms are involved in microbial killing [18], chemotaxis of neutrophils [16,19], and a variety of signal transduction cascades in response to growth factors relevant for the abovementioned processes of cutaneous wound repair [20]. However, this is an emerging field and data specifically concerning the role of NOX enzymes in cutaneous wound repair are limited. The following sections provide an overview of the current understanding of the role of ROS and redox signaling in the abovementioned phenomena known to play a role in wound repair, mainly relying on data obtained in research fields other than cutaneous wound repair. Furthermore, we will address their role in the development of chronic wounds and discuss potential therapeutic implications.

## 2. The Role of ROS and Redox Signaling during the Coagulation Phase

Blood coagulation through activation and recruitment of platelets following vessel-wall injury occurs concomitantly with a sharp increase of ROS production at the wound site [21], suggesting that redox signaling regulates wound repair as early as during the coagulation phase [11]. When active tissue factor is exposed to blood after injury, the coagulation cascade is initiated, leading to the formation of thrombin. Besides its well-known role in the coagulation cascade, thrombin induces ROS generation by NOX enzymes in vascular cells, which subsequently promote a thrombogenic cycle via upregulation of tissue factor expression [22,23]. Consistently, H_2_O_2_ has been shown to activate latent cell surface tissue factor on vascular smooth muscle cells (VSMCs) [24]. Platelets have been shown to produce ROS [25], and the upregulation of tissue factor expression by activated platelets is mediated through ROS generation by NOX enzymes in VSMCs [19,26]. When platelets come into contact with collagen they start producing ROS in a NOX-dependent manner, which results in increased platelet recruitment [27].

Platelet-derived growth factor (PDGF), released by platelets upon activation, plays an important role in the recruitment and proliferation of various cells during wound repair and is dependent on H_2_O_2_ [28]. When PDGF binds to its receptor, H_2_O_2_ is produced in non-phagocytic cells in a Rac1 dependent manner [29]. Rac is a small GTPase of the Rho family and it has been shown to play an important role in the regulation of NOX1 and NOX2, possibly also of NOX3 and NOX4 [8,20]. This is of particular interest given that recombinant PDGF (PDGF-BB or Becaplermin) is currently the only growth factor approved by the Food and Drug Administration for the treatment of chronic wounds [30]. Sufficient oxygenation of the wound might be required to make the wound receptive to PDGF-BB treatment.

## 3. The Role of ROS and Redox Signaling during the Inflammation Phase

It is clear that ROS production during inflammation occupies a central role as a direct germicide used by neutrophils and macrophages. The isoform NOX2 plays a predominant role in microbial killing as it is responsible for producing large amounts of ROS in what is called the respiratory burst [10,18]. Inside phagosomes, high concentrations of ROS create a toxic oxidative stress environment for phagocytozed microbes leading to DNA damage, lipid peroxidation and oxidation of amino acids [31]. Patients with chronic granulomatous disease, a rare congenital abnormality of the phagocyte NOX2 system, show impaired wound repair and susceptibility to wound infection [32,33]. Besides ROS, reactive nitrogen species (RNS) contribute to microbial killing. RNS are produced following the reaction of nitric oxide (NO) with superoxide resulting in highly reactive species than can directly interact with various biological targets [34].

Besides direct microbial killing, ROS are emerging as central signaling molecules modulating the inflammatory response. Depending on its concentration and via NF-κB signaling cascades, H_2_O_2_ can either induce a pro-inflammatory control loop that increases pathogen removal or an anti-inflammatory control loop, which avoids an exacerbated harmful inflammatory response [35]. Other compelling examples for the role of ROS in the regulation of inflammation are that H_2_O_2_ in low concentrations induces neutrophil chemotaxis [36], and that the overexpression of thioredoxin, a ROS degrading protein, suppresses leucocyte recruitment [37]. Furthermore, H_2_O_2_ and superoxide modulate leukocyte adhesion molecule expression and leukocyte endothelial adhesion [38]. ROS induce spreading of macrophages via extracellular signal-regulated kinases [39]. Monocytes are activated when they adhere to the extracellular matrix by their specific integrin receptors. This adhesion can be induced by H_2_O_2_ in vitro [40]. TNFα and IL-6 induce neutrophil and macrophage migration and facilitate phagocytosis. Their biosynthesis has been shown to be ROS-inducible [41]. IL-6 plays an essential role in skin wound repair, as evidenced by delayed wound healing in IL-6-deficient mice [42].

The isoform NOX4 might play a role in phagocyte recruitment as NOX4 deficiency is associated with a decrease in phagocytic cell presence. In our laboratory, we have observed that NOX4 deficiency leads to a decreased expression of NOX2 during cutaneous wound repair, implying a role for NOX4 in phagocytic cell recruitment [43]. This recruitment could take place through NOX4 dependent IL-6 expression, similar to what has been shown in human microglia and non-small cell lung cancer cells [44,45].

## 4. The Role of ROS and Redox Signaling during the Proliferation Phase

### 4.1. Angiogenesis

It is increasingly accepted that redox-signaling plays a central role during angiogenesis [7]. H_2_O_2_ in low concentrations facilitates angiogenesis in wound repair in mouse wound models, which can be reversed by adenoviral gene transfer of catalase, a H_2_O_2_-decomposing enzyme [11]. The notion that redox signaling plays an important role in angiogenesis is supported by evidence suggesting that a large number of antioxidants limit angiogenesis [7]. This includes Vitamin C [46], quercetin [47], resveratrol [48], and several others. The suggested underlying mechanisms include the downregulation of nitric oxide synthase (NOS) expression and activity [49], and interference with vascular endothelial growth factor (VEGF) signaling [46].

Angiogenesis is a complex process that requires a solid, fibrin-rich extracellular matrix, as well as migratory and mitotic stimulation of endothelial cells [50]. Many soluble factors are involved in angiogenesis, including VEGF [51], fibroblast growth factors (FGFs) [52], PDGF [53], angiopoietin [54] and many others. The expression of VEGF increases within hours after keratinocytes, fibroblasts, and endothelial cells at the wound site are exposed to hypoxia [55,56,57]. There are numerous studies showing that ROS are involved in VEGF and VEGF receptor signaling. H_2_O_2_ has been reported to potently stimulate VEGF expression in keratinocytes [58] and macrophages in culture [59]. ROS not only increase VEGF transcription but also increase VEGF mRNA stability and enhance VEGF release [60]. The theory of an important role of ROS in VEGF-induced angiogenesis is further strengthened by the presence of specific mechanisms insuring the function VEGF in an environment of oxidative stress. When the amount of ROS increases, proteins are at risk of oxidative damage [61]. VEGF is protected from this damage by the extracellular chaperone glypican-1, which can restore the receptor-binding ability of oxidation-damaged VEGF [62,63].

Besides inducing the expression of VEGF, ROS seem to be involved in VEGF downstream signaling. VEGF induces ROS production in endothelial cells by a Rac1-regulated NOX-dependent mechanism [64]. Furthermore, it has been reported that intracellular ROS is required for the activation of the transcription factor NF-κB, which in turn is needed for VEGF-mediated vascular smooth muscle cell migration [65]. The VEGF-induced ROS production is inhibited by the NOX inhibitor diphenylene iodonium, as well as by overexpression of dominant-negative Rac1, suggesting that VEGF induces NOX-mediated ROS production [66].

It seems that NOX enzymes are the major source of ROS during wound angiogenesis and that they are produced by a wide range of cells, including inflammatory cells, vascular endothelial cells, fibroblasts and epithelial cells [11,67]. It is thus conceivable that NOX enzymes are critical for angiogenesis. Consistently, NOX1 has been reported to be a potent trigger of the angiogenic switch in tumors. NOX1 strongly upregulates VEGF mRNA both in cultured fibroblasts and epithelial cells, and VEGF receptors are highly induced in vascular cells in NOX1-expressing tumors. The angiogenic properties of NOX1 are reversed when cells co-express catalase, suggesting that NOX1-mediated ROS are essential for the angiogenic switch [68]. As mentioned above, Rac1 is an import regulator of NOX-mediated redox signaling. It has been shown that Rac1 gene transfer accelerates wound contraction and closure in a mouse model, and that this is associated with a significant increase in VEGF expression. In vitro, Rac1 overexpression strongly induced VEGF expression in human keratinocytes [58]. The effects of VEGF on endothelial cell proliferation and migration are dramatically inhibited in cells transfected with NOX2 antisense oligonucleotides, suggesting that NOX2-derived ROS play an important role in angiogenesis in vivo [66].

Hypoxia inducible factor 1 (HIF1) is a primordial player in the cell response to hypoxia [69]. Under normoxic conditions, HIF1α is constantly degraded through hydroxylation, a process that is strictly oxygen-dependent. Under hypoxic conditions, HIF1α escapes degradation and forms a stable dimer with HIF1β, the active form of the transcription factor [70]. HIF1 is a potent inducer of VEGF [71], inducing angiogenesis and other important processes in wound repair. VEGF regulates vascular endothelial cell migration, proliferation and permeability, and functions as an anti-apoptotic factor for newly formed blood vessels [72].

NOX4 could play a key role in cell responses to hypoxia during wound repair. Pulmonary artery smooth muscle cells cultured under hypoxic conditions showed increased expression of NOX4 [15,73] and their proliferation was NOX4-depended [74]. There is increasing evidence that HIF stabilization can also be induced directly by ROS [75,76]. In fact, there appears to be a positive feed-forward loop involving NOX4 and HIF1: ROS generated by NOX4 activate HIF-1α [77] and HIF-1α activates the expression of NOX4 [78]. However, it has been shown that ROS stimulation of VEGF is HIF-independent [58]. In a recent study, we observed that HIF1α and CD31 expression during cutaneous wound repair were significantly weaker in NOX4 knockout mice suggesting that NOX4 is involved in HIF1α expression and angiogenesis during wound repair [43]. This is in line with earlier studies that showed that NOX4 is required for effective angiogenesis [79]. NOX4 has also been extensively studied for its role as an oxygen sensor, and it has been demonstrated that NOX4 is capable of generating hydrogen peroxide as a function of oxygen concentration throughout a physiological range of pO_2_ values [80].

NO is a free radical gas that has been known since the 1970s for its role in vasodilation, also playing an important role in platelet activation and vascular cell signaling. Cutaneous wound repair is associated with a significant increase in the expression of gens that regulate the production of NO, including the inducible nitric oxide synthase (iNOS) [81,82]. High levels of NO are associated with the stabilization of HIF1α and thus mimic a hypoxic state under normoxic conditions [83]. Consistently, it has been reported that NO triggers VEGF expression in cultured keratinocytes and during wound repair [84].

### 4.2. Re-Epithelialization

Besides wound contraction, re-epithelialization is an important process for effective wound closure. Wound re-epithelialization relies on the migration, proliferation and differentiation of keratinocytes at the wound edge and at any transected wound appendages (hair follicles, sweat ducts) [85]. Recent data suggest that epithelial cells are capable of regulated ROS production via NOX complexes [86] but this is an emerging field of research. Recent data also suggest that keratinocytes produce ROS via NOX enzymes, including NOX4 [86,87]. It has been shown that ROS at low concentrations induces keratinocyte migration in vitro [88,89]. The isoform NOX1 has been studied in epithelial repair of intestinal mucosa healing and it has been reported that NOX1 mediates epithelial migration through activation and modification of focal adhesion proteins involved in regulating cell migration [90]. IGF-1 plays an important role in epidermal keratinocyte migration by facilitating membrane protrusion via activation of Rho family proteins [91]. It has been shown that the regulation of the structure and function of IGF-1 is redox-sensitive [92,93]. Other sources of ROS involved in re-epithelialization include xanthine oxidoreductase, which has the unique capacity to produce both ROS and NO and has been reported to stimulate keratinocyte proliferation and angiogenesis in a mouse model of skin wound repair [94].

Migrating keratinocytes dissect the wound, separating eschar from viable tissue, producing MMPs and activating plasmin by plasminogen activators [95]. MMP-1 is an important constituent of the matrix-degrading apparatus of keratinocytes and is expressed in a NOX4-mediated, ROS-dependent way [96]. MMP-2 and MMP-9 also contribute to keratinocyte migration since their inhibition seems to hinder migration [97,98]. It has been shown that H_2_O_2_ activates MMP-2 through the NF-κB pathway [99]. Furthermore, the oxidative modification of fibrin by ROS is specific and favors fibrinolysis [100].

Besides migration, the continuous proliferation of epidermal keratinocytes is crucial for providing a sufficient supply of migrating cells during re-epithelialization. EGF, TGFα and KGF promote this process. H_2_O_2_ plays a central role in the regulation of EGF signaling and receptor phosphorylation [101,102]. Keratinocytes bordering the defect and in hair follicles show high expression of TNFα [103], which seems to have an autocrine stimulating effect [104]. TNFα stimulation leads to restructuring of the cytoskeleton of keratinocytes, an oxygen-dependent process that is initiated within hours after injury [105]. ROS induce TGFα expression in fibroblasts suggesting that oxidative stress in fibroblasts in the granulation tissue could contribute to re-epithelialization [106]. KGF plays an important role as a stimulator of keratinocyte proliferation. It has been reported that transfected keratinocytes and fibroblasts stably expressing KGF, applied on a membrane carrier to burn wounds in pigs, significantly increases re-epithelialization [107], and it has been reported that ROS can directly trigger the activation and internalization of the KGF receptor [108].

### 4.3. Wound Contraction

In order to guarantee rapid wound closure, wound contraction is of utmost importance. When the wound contracts, the uninjured skin surrounding the wound is pulled into the defect, significantly reducing the amount of time and tissue needs to reestablish the integrity of the skin barrier after injury [109]. There are still some open questions regarding the exact mechanisms that lead to the generation of contractile forces within the wound bed. There seem to be two major mechanisms of wound contraction: (1) organization and stiffening of the ECM [110] and (2) cellular contraction of myofibroblasts [111,112]. Myofibroblasts are specialized contractile fibroblasts largely responsible for ECM remodeling and contraction in mature granulation tissue [113]. Myofibroblast differentiation occurs in response to growth factors and mechanical stress and is characterized by the formation of large stress fibers and focal adhesions [111,112,114,115]. One prominent feature of myofibroblast differentiation is the expression of α-smooth muscle actin (α-SMA) in stress fibers, and this is considered to be the basis for their high contractility [111,112].

**Figure 1:**
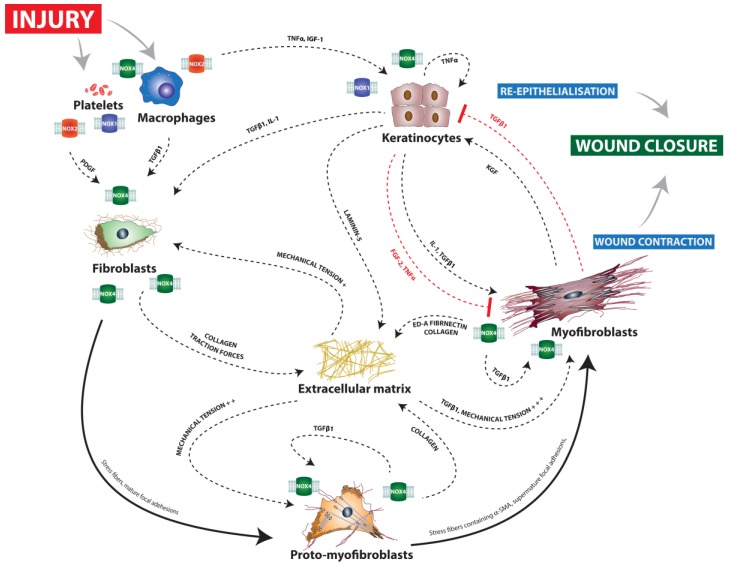
Schematic view of the interactions underlying wound closure and major implications of NOX enzymes. Solid arrows represent differentiation processes, dashed arrows promoting interactions and red dashed T arrows inhibitory interactions. After injury, the activation of platelets and macrophages leads to the production of large amounts of ROS. NOX2 and NOX1 play a major role in these processes. NOX4 has also been suggested to play a role in macrophage recruitment. Growth factors released by platelets and macrophages trigger the proliferation and migration of keratinocytes, leading to re-epithelialization. NOX1 and NOX4 possibly play a direct role in keratinocyte migration. Different precursor cells and fibroblasts are attracted to the wound site by growth factors including TGFβ1 and PDGF, a process that is likely to be mediated by NOX4. Migrating fibroblasts stiffen the extracellular matrix (ECM) by producing collagen and inducing traction forces due to their migratory activity. Also, NOX4 seems to play a crucial role here. The stiffened ECM induces the differentiation of fibroblasts into moderately contractile proto-myofibroblasts. Proto-myofibroblasts produce large amounts of ECM components, including mostly collagen, further stiffening the ECM, a process that has been shown to be dependent on NOX4. In addition, they release TGFβ1 from dormant ECM deposits via integrin expression. Induced by the further increased mechanical tension of the ECM, highly contractile myofibroblasts develop, characterized by de novo expression of αSMA, which enables them to generate greater contractile forces. The role of NOX4 in cutaneous myofibroblast differentiation is controversial as it has been shown that NOX4 is a prerequisite for myofibroblast differentiation during pulmonary fibrosis but does not seem to be required during cutaneous wound repair.

Myofibroblast differentiation can be understood as a two-step process in which fibroblastic progenitor cells (e.g., fibrocytes [116], mesenchymal stem cells [117], epithelial cells [118], and perivascular precursor cells [119]) differentiate in a first step into moderately contractile α-SMA-negative proto-myofibroblasts. Triggered by further stiffening of the ECM, as well as by growth factors (mostly TGFβ1, but also PDGF) [120,121,122], proto-myofibroblasts then further differentiate into highly contractile α-SMA-positive myofibroblasts [123,124] (Figure 1).

Fibroblastic progenitor cells differentiate into proto-myofibroblasts in response to mechanical stress, expressing β- and γ-actin-containing stress fibers that terminate in focal adhesions [111,125]. As proto-myofibroblasts contract, they further contribute to a stiffening of the ECM, which makes them even more contractile and, together with the increased availability of TGFβ1, eventually leads to their differentiation into myofibroblasts [126,127].

Our knowledge concerning the role of ROS and NOX-mediated redox signaling in myofibroblast activity is still limited and relies mainly on studies conducted in fields other than skin wound repair. NOX2 might play a role in myofibroblast differentiation, as a recent study found that oxidants produced by NOX2 mediate *α*-SMA and ECM upregulation in human dermal fibroblasts in response to TGFβ1 [128] Recently, the role of NOX4 in bleomycin-induced pulmonary fibrosis has been investigated [129]. The accumulation of myofibroblasts is a hallmark of advanced and progressive pulmonary fibrosis. This accumulation is mainly driven by TGFβ1 signaling [130,131], in line with what has been observed in cutaneous wound repair studies [115]. Activation of transcription factors of the Smad family, in particular of Smad2 and Smad3, is one of the major TGFβ1-dependent signals [131,132,133]. This activation occurs through phosphorylation by the TGFβ1 receptor [134]. NOX4-deficient mice show significantly reduced phosphorylation of Smad2 and α-SMA expression, suggesting that NOX4 is necessary for TGFβ1-induced myofibroblast differentiation [129]. TGFβ1 induces NOX4 expression in cultured pulmonary smooth muscle cells [135], and NOX4 enzymes influence myofibroblast differentiation of cardiac fibroblasts [136]. It has been reported that TGFβ1 induces H_2_O_2_ production in human fibroblasts [137]. ROS produced by NOX4 were shown to be a prerequisite for TGFβ1-induced myofibroblast differentiation, ECM production and contractility in lung-derived fibroblasts [138]. These recent findings suggest that NOX4-mediated redox signaling could be involved in (myo) fibroplasia during skin wound repair.

However, in a recent study we found that NOX4 was not required for myofibroblast differentiation during skin wound repair in a mouse model [43]. We found that NOX4 deficiency had no impact on myofibroblast expression but led to significantly impaired wound contraction, revealing a dissociation of myofibroblast expression and wound contractility. We also observed that hyperbaric oxygen therapy (HBOT) significantly accelerates wound contraction in rats, but does not increase fibroblastic cell recruitment or myofibroblast differentiation. Of note, we observed a significant increase in collagen deposition during early time points in wounds exposed to HBOT compared to a standard wound dressing only [139]. These findings raise questions regarding the role of the myofibroblast as the main cell orchestrating wound contraction and the exact mechanisms that lead to the generation of contractile forces in the wound bed. In fact, there is increasing evidence suggesting that αSMA expression is not a prerequisite for myofibroblast contraction during wound repair and that smooth muscle γ-actin and skeletal muscle α-actin can compensate for a lack of αSMA [140,141]. It is possible that NOX4 plays a role in the expression of muscle actins other than αSMA, such as smooth muscle γ-actin or skeletal muscle α-actin, and that this is responsible for myofibroblast function during wound repair. If this is the case, NOX4-deficient myofibroblasts could be less contractile despite expressing normal amounts of αSMA.

The mechanical properties of the ECM are of great importance for translating the cellular contraction of myofibroblasts into contraction of the wound tissue. While the provisional matrix of early granulation tissue is highly compliant, mature granulation tissue becomes increasingly stiff over time [142]. It is currently thought that traction forces induced by fibroblast migration within the fibrin-rich provisional matrix and their ECM-remodeling activity gradually increases the stiffness of granulation tissue [110,111,143,144]. Migrating fibroblasts remodel the ECM, synthesizing a variety of ECM components, such as collagen (predominantly types I and III) and fibronectins, various MMPs, and tissue inhibitors of metalloproteinases [127,145]. NOX4 has been shown to be required for ECM production in lung-derived fibroblasts in vitro [138,146]. Targeting NOX4 results in the attenuation of an established fibrotic response, with a reduction in gene transcripts for the extracellular matrix components collagen 1α1, collagen 3α1, and fibronectin [146]. Accordingly, we recently found that collagen deposition was significantly reduced in NOX4-deficient mice [43]. A less dense collagen matrix could explain a lack of translation of the cellular forces into a contraction of the tissue as a whole.

The hydroxylation of proline is oxygen-dependent and pro-collagen molecules require hydroxyproline to form stable triple helices [1]. In vitro studies found that prolyl hydroxylase needs 20 mmHg O_2_ to function at 50% enzymatic speed [147], and fibroblast cultures revealed that a minimum of 30–40 mmHg O_2_ is needed for collagen synthesis [148]. A study of collagen deposition in patients’ wounds revealed that the amount of collagen deposited was directly proportional to tissue oxygen tension [149].

In addition to the expression of ECM components, their crosslinking is likely to play an important role in the transduction of contractile forces [142,150,151]. This is a little understood process where NOX enzymes are particularly likely to play a crucial role. NOX-mediated dityrosine crosslinking of the ECM is a possible player in wound contraction. In fact, we recently found that dityrosine formation significantly increased during the wound repair process, suggesting it has a physiological role [43]. NOX4-deficiency leads to significant impairment of dityrosine expression, providing a possible explanation as to how myofibroblast contraction in NOX4-deficient mice might be less effective in contracting the wound. It is conceivable that myofibroblast contraction is only effectively translated into tissue contraction if the cells are embedded in a well cross-linked ECM [42,44,45].

Tyrosine dimerization is thought to require the presence of ROS (i.e., H_2_O_2_) and a peroxidase [152,153]. In fact, there is strong evidence that peroxidases mediate ECM crosslinking [154,155]. One possible source of peroxidase during physiological wound repair is macrophages and neutrophils, as they are known to secrete myeloperoxidase (MPO), which is mainly known for its role in host defense. However, in our study we observed no correlation between dityrosine formation and MPO expression. Also, there was no difference in expression between wildtype and NOX4^−/−^ mice, suggesting that MPO is not the main peroxidase catalyzing dityrosine formation during wound repair.

A promising candidate for a major role in mediating ECM crosslinking is vascular peroxidase 1 (VPO1, a.k.a. peroxidasin, PXDN) as it contains, besides its peroxidase domain, modules that are characteristic of the ECM [156]. VPO1 has also been shown to be secreted by myofibroblasts in the ECM, where it organizes into a fibril-like network co-localizing with fibronectin [157]. In fact, Lázár et al. found that VPO1 mediates the crosslinking of collagen IV in hot spots near the cell surface [158]. It has also been suggested that VPO1 catalyzes tyrosyl radical formation and promotes dityrosine cross-linking [159]. VPO1 requires H_2_O_2_ to function, which is supplied to the enzyme by a currently unknown cellular source. In a model of hypoxia-induced pulmonary hypertension, Liu et al. propose that NOX4 is a provider of hydrogen peroxidase for VPO1 during inflammatory reaction [160], but the relationship between NOX4 and VPO1 is yet to be defined. We believe NOX4 is likely to be a promising candidate as a provider of H_2_O_2_ for VPO1-mediated collagen crosslinking through tyrosine dimerization, which could be an important mechanism of granulation tissue stiffening during skin wound repair. Other possible catalyzers of tyrosine cross-linking during wound repair are DUOX enzymes as they present a NOX domain as well as an extracellular peroxidase domain and have been reported to be involved in ECM crosslinking in nematodes [154]. Further research should be done in order to understand whether DUOX enzymes also play a role in ECM crosslinking during wound repair in higher animals.

Besides tyrosine formation, peroxidases catalyze other protein cross-links, which might also participate along with dityrosine in the stiffening of the ECM. For instance, peroxidase-catalyzed cross-links are formed from the deamination of protein lysyl ɛ-amino groups to form lysyl aldehydes, which then react with amino acid residues of adjacent molecules [161,162]. Collagen cross-linking mediated by lysyl oxidase expressed by fibroblasts has been suggested as a possible mechanism of tissue stiffening in early granulation tissue [163].

It has been suggested that NO is produced by fibroblasts and serves as an autocrine regulator of collagen production and cell contraction [164]. In fact, the blockade of NO synthesis impairs wound repair [165] and is associated with reduced collagen deposition and decreased mechanical resistance of wounds in rats [164].

## 5. The Role of ROS and Redox Signaling during the Maturation Phase

After wound closure, controlled cell removal and ECM remodeling are crucial to terminate repair. Myofibroblasts, as well as vascular and inflammatory cells, physiologically disappear by apoptosis and proteolytic enzymes (mainly MMPs) secreted by macrophages, epidermal cells, endothelial cells, and fibroblasts degrade ECM components [166,167,168,169]. Cell persistence can lead to hypertrophic scarring, associated with increased levels of TGFβ1, α-SMA expression, ECM deposition, and vascularization [170,171]. Clinically, a thickening of the scar tissue is observed, while progressive contraction and dysesthesia lead to functional disabilities. The role of ROS and redox signaling in scar maturation has not yet been specifically investigated to our knowledge until now, but there are extensive data from other fields suggesting a major role in key processes such as apoptosis and ECM remodeling.

The role of NOX enzymes in apoptosis has been intensively investigated, especially in the fields of cancer and cardiovascular research, where strong implications of NOX enzymes in the regulation of apoptosis have been reported [172]. However, the data are contradictory and the exact mechanisms by which NOX-mediated ROS production either promotes or prevents apoptosis remain unclear. The current understanding is that NOX enzymes have both pro-survival and pro-apoptotic properties depending on the concentration and duration of expression [173,174]. A detailed discussion of this subject would, however, go beyond the scope of the current review. Of interest for the field of wound repair, NOX-mediated ROS production has been repeatedly associated with different fibroblast growth factor signaling pathways and is likely to play a direct role in fibroblast apoptosis during scar maturation [175]. FGF2 has been shown to significantly increase myofibroblast apoptosis and decreases mature collagen bundle formation [176,177]. Basic FGF seems to reduce granulation tissue volume and has been suggested as a possible target for promoting scarless wound repair through the induction of myofibroblast apoptosis [178,179].

Besides apoptosis, the remodeling of the ECM is an important feature of the maturation phase, leading to a progressive strengthening of the scar tissue. Collagen type III, which is predominant during the proliferation phase, is gradually replaced by collagen type I, and the fibers are increasingly crosslinked and oriented [180]. As discussed above, NOX enzymes play an important role in the synthesis as well as the crosslinking of collagen fibers. Effective remodeling of the ECM relies heavily on MMPs, which catalyze the hydrolysis of major ECM molecules, including collagen, elastin, laminin, and fibronectin [180]. NOX-mediated ROS have been reported to stimulate the expression of MMP-1 and MMP-9, both highly active during scar maturation [181].Of interest, it has been recently shown that NOX2 siRNA suppresses collagen production in primary keloid dermal fibroblasts, suggesting that NOX2 plays a causal role in the overproduction of collagen in keloids [128].

## 6. ROS and Redox Signaling in Chronic Wounds

Understanding the pathogenesis of chronic wounds has proven to be very difficult. The complexity of the wound repair process, the heterogeneity of chronic wounds, the difficulties in performing clinical trials, and the lack of animal models have hampered progress in elucidating the pathophysiology of chronic wounds. Ischemia (including reperfusion injury), diabetes, chronic inflammation, and age-related cell senescence are the most important factors favoring the development of chronic wounds.

Chronic wounds are in the great majority of cases associated with a chronic state of inflammation [182]. It has been shown that the proliferation phase can only effectively be initiated if the number of macrophages diminishes. If macrophages persist, the inflammatory phase is prolonged and the proliferation phase delayed [183]. Chronic inflammation also induces cell senescence, which is increasingly recognized as being one of the key pathophysiological phenomena in the development of chronic wounds [184], especially with regard to fibroplasia [185].

As discussed above, ROS play an important role in wound repair via redox signaling. However, ROS in excessive amounts have a deleterious effect, inducing lipid peroxidation, protein modification and DNA damage leading to apoptosis and senescence [18]. High levels of ROS occur if either their production is increased or their detoxification impaired. The majority of ROS during wound repair are most likely being produced by neutrophils and macrophages during the inflammatory phase [184]. As chronic wounds enter a state of chronic inflammation, the amount of ROS remains high over a prolonged period of time, with subsequent cell damage further accelerating inflammation [184]. In fact, oxidative stress in chronic wounds seems to be dramatically increased when compared to acute wounds [186]. High concentrations of ROS have a toxic effect, as shown in severe endothelial damage in wounds in mice that lack peroxiredoxin-6, a ROS-detoxifying enzyme [187].

The senescence of fibroblasts is increasingly discussed as a hallmark in the development of chronic ulcers. Oxidative stress has been suggested to be one possible explanation of how fibroblasts could enter a senescent state in chronic wounds [188], as high levels of oxidative stress lead to premature senescence in fibroblasts in culture [189,190]. Indeed, fibroblasts isolated from the margins of chronic wounds show signs of premature senescence [185,191]. Of note, NOX1 has been suggested to be involved in p53- and Rb-dependent fibroblast senescence [192].

Following tissue injury, the microenvironment of the wound suffers a dramatic drop in oxygen supply due to vascular disruption. This drop in oxygen tension is aggravated by high oxygen consumption by inflammatory and reparative cells in the wound [193]. Despite the hypoxic environment in the early course of wound repair, endothelial cells and fibroblasts exhibit enhanced rates of migration, protein synthesis, and proliferation [194]. In fact, acute hypoxia stimulates key components of wound repair [6]. Endothelial cells produce increased amounts of endothelial adhesion molecules that stimulate extravasation and tissue invasion by neutrophils and macrophages [195]. In addition, keratinocyte proliferation and migration are stimulated by acute hypoxia [196,197]. Wound scratch assays have revealed that human dermal keratinocytes show increased migration and MMP-2 production under acute conditions of hypoxia [97]. Cultured dermal fibroblasts react to acute hypoxia with upregulation of TGFβ1, collagen I, and VEGF secretion and increased proliferation and migration [198,199,200,201].

During physiologic wound repair, oxygen levels are gradually re-established by vasodilation, increased vascular permeability, and angiogenesis. If these processes fail or if the wound is located in a setting of chronic ischemia, the wound repair process is delayed or stagnates. In fact, chronic ischemic wounds are typically stuck in an inflammatory state, failing to enter the proliferation phase, showing poor proliferation and matrix deposition [202]. In contrast to the stimulating effect of physiological acute hypoxia, persistent hypoxia has a strong inhibitory effect on some key features of the proliferative phase of wound repair [202]. In a rat model of ischemic cutaneous wounds developed in our laboratory, chronic ischemia significantly delayed wound closure. The delay in wound closure was associated with a significant decrease in granulation tissue formation, myofibroblast expression, and wound contraction. Myofibroblasts appeared late and their peak expression was significantly reduced [203]. These findings suggest that decreased wound contraction plays an important role in delayed ischemic wound repair, due in part to impaired myofibroblast development and activity.

In vitro experiments performed in our laboratory revealed that when dermal fibroblasts are cultured under conditions of persistent hypoxia, expression of myofibroblast function is significantly reduced [201]. Culturing dermal fibroblasts on deformable silicone substrates and quantifying wrinkling revealed that persistent hypoxia significantly impairs cell contraction. The decrease in myofibroblast expression and contraction was associated with an increase in TGFβ1 secretion but a downregulation of the TGFβ1 receptor suggesting that hypoxia has a TGFβ1-desensitizing effect on fibroblasts [201]. This is in line with the findings of other groups that have associated altered TGFβ1 signaling with impaired wound repair in ischemic conditions [6,204,205,206]. Another study showed that dermal fibroblasts cultured under conditions of persistent hypoxia had decreased levels of collagen synthesis [199].

It is still unclear how persistent ischemia interferes with TGFβ1 signaling. One possibility is that NOX-mediated redox signaling plays a role in these phenomena. NOX4 has been reported to be upregulated in hypoxia in an HIF-dependent manner [78]. There is some evidence suggesting that persistent ischemia provokes a downregulation of HIF1 [207]. NOX4 seems to be required for TGFβ1-mediated angiogenesis [79] and fibroplasia [129]. It is tempting to speculate that HIF1 downregulation induced by persistent ischemia results in reduced expression of NOX4, which in return decreases TGFβ1-mediated fibroplasia and angiogenesis.

Diminished perfusion leading to local hypoxia plays a role in all types of chronic wounds. The repercussions of a lack of oxygen are much more complex than a simple reduction in metabolism. The duration of hypoxia and the switch between hyperoxia and hypoxia, as is frequently seen in chronic wounds, lead to a series of complex changes in cell behavior. The effects of hypoxia are sometimes counterintuitive, such as hypoxia leading to increased ROS production. 

Oxidative stress appears to be increase under conditions of ischemia, as hypoxia has been shown to induce ROS production in vitro [3,208]. Cyclic episodes of ischemia and reperfusion occur when patients with venous insufficiency, arteriosclerosis, and diabetes mellitus change position relative to gravity. Ischemia–reperfusion injury is likely to play an important role in the development of chronic wounds [209]. With each reperfusion, additional neutrophils flood into the wound site and migrate through the activated endothelium, further contributing to an vicious inflammatory cycle [210]. In addition, temporary reperfusion delivers new oxygen to the wound site, which could lead to high levels of ROS, possibly contributing to tissue damage [211]. Animal models have shown that repetitive ischemia reperfusion cycles have a deleterious effect on wound repair [212]. Of note, repeated ischemia–reperfusion events delay wound healing more than prolonged periods of ischemia [213]. Recently, it has been shown in a model of myocardial ischemia–reperfusion injury that NOX1 and NOX2 play an important role in reperfusion injury as knockout mice showed a significantly smaller infarct size [214]. Another important source of ROS during ischemia–reperfusion injury is xanthine oxidoreductase, which has been extensively studied over the last decades. It has been reported repeatedly that the inhibition of xanthine oxidoreductase has a beneficial effect on ischemia–reperfusion injury but there are still some controversies, especially as there seems to be a tissue-specific effect [215].

**Figure 2:**
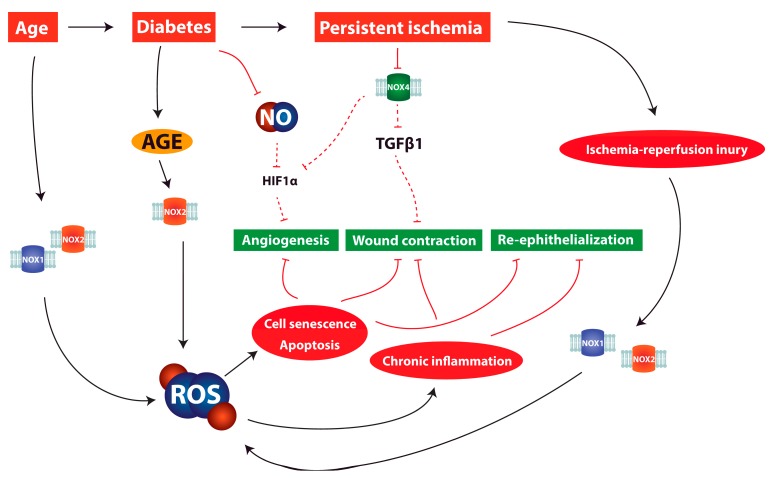
Illustration of pathomechanisms involving NOX enzymes and reactive oxygen species (ROS) in chronic wounds. Solid black arrows represent promoting interactions, red solid T arrows direct inhibitory interactions and red dashed T arrows indirect inhibitory interactions. Aging, diabetes, and persistent ischemia are associated with excessive ROS production. NOX1 and NOX2 have been reported to be responsible for excessive ROS production during ischemia–reperfusion injury. Through the production of advanced glycation end products (AGE), hyperglycemia triggers an overproduction of ROS. Excessive oxidative stress leads to cell senescence, apoptosis, and chronic inflammation, hampering key processes of wound repair. In addition, persistent ischemia is likely to lead to impaired NOX4 activity, directly interfering with TGFb1 signaling and leading to decreased myofibroblast contractility. Reduced NOX4 activity also seems to reduce HIF1a signaling, making effective angiogenesis impossible and further aggravating the ischemic condition. Diabetes has been shown to lead to a reduced availability of NO, facilitating the destruction of HIF1 and hindering angiogenesis.

It is an established fact that angiogenesis is impaired in diabetic patients [216]. Hyperglycemia has been repeatedly associated with endothelial cell dysfunction, provoking an imbalance between vasocontricting and vasodilating substances [217,218,219]. Through the creation of advanced glycation end products (AGE), hyperglycemia triggers an overproduction of ROS [219], hampering vasodilation by limiting the availability of NO [220]. In fact, impaired diabetic wound repair is associated with decreased NO synthesis [221]. During hypoxia, low concentrations of NO facilitate the destruction of HIF1α and thus impair HIF signaling [83] (see Figure 2). Animal studies suggest that l-arginine supplementation, a substrate for NO synthesis, has a beneficial effect. In fact, the impairment of NO synthesis in diabetic rats could be partly reversed and wound repair capacities enhanced by l-arginine [222]. The exogenous application of NO in hydrogels could partly reverse the reduced collagen production and mechanical strength of the wound associated with reduced NO production in diabetic rats [223,224].

Oxidative stress induced by hyperglycemia has been suggested as a mechanism that may exacerbate tissue injury after stroke and myocardial infarction [225,226]. Both ischemia (–reperfusion injury) and hyperglycemia increase production of ROS and it is conceivable that the two factors accelerate each other. Ischemia leads to oxidative stress through the generation of ROS by NOX [227], while glucose was reported to be a prerequisite for reperfusion-induced superoxide production by NOX [225]. Hyperglycemia can increase the assembly of NOX enzymes and therefore enhance ROS production [228], and leads to the generation of ROS through the accumulation of AGE [229,230] (see Figure 2). Insulin resistance, and the associated increased insulin levels, might also contribute to excessive ROS production in diabetic wounds, as insulin has been reported to induce NOX-produced ROS in human skin fibroblasts ex vivo [231].

Under hyperglycemic conditions, the combination of oxidative stress, acidosis, and hypercoagulability creates a NOXious cellular environment that favors progressive ischemic necrosis. As the mechanisms through which both ischemia and hyperglycemia induce cellular toxicity are similar, it is possible that they amplify each other. We suggest that oxidative stress provoked by ischemia and exacerbated by hyperglycemia constitutes a key mechanism in the pathophysiology of ischemic cell necrosis.

Age plays a predominant role in the pathophysiology of chronic wounds; the majority of chronic wounds occur in people aged 60 or over [191,209]. Cell senescence, defined as a state of diminished metabolism and reduced mitogenic activity, alter patterns of gene expression and decrease responsiveness to growth factors, and thereby reduce repair capacity in aged individuals [6,232]. Aged human fibroblasts are more susceptible to oxidative stress-induced senescence and cell death [233]. They show unresponsiveness to TGFβ1 and reduced migration and proliferation [205,234]. Aged fibroblasts show an increased production of MMPs and a decreased level of MMP-inhibitors, which could further impair granulation tissue formation in aged individuals [235]. While young keratinocytes respond to acute hypoxia with proliferation and migration, aged keratinocytes show impeded responses to hypoxia [196]. In cell cultures, it has been shown that smooth muscle cells of aged rabbits express lower levels of VEGF in response to hypoxia [236]. Type I and type II collagen synthesis are reduced in aged fibroblasts associated with degraded collagen accumulation, leading to decreased biomechanical properties [237]. ROS and oxidative stress have long been linked to aging and diseases prominent in the elderly such as hypertension, atherosclerosis, diabetes, and atrial fibrillation, and NOX enzymes are believed to play a major role in age-related increase in in oxidative stress [238]. NOX-induced cellular aging is likely to play a role in the development of chronic wounds but to discuss it in detail would go beyond the scope of the current review; details can be found elsewhere [239].

## 7. Discussion and Clinical Relevance

As a logical consequence of the observations that ischemia results in impeded wound repair and that virtually all chronic wounds show some degree of impaired wound oxygenation, the therapy of chronic wounds with either topically or systemically administered oxygen has a long history [240]. Many experimental studies have since provided evidence that increasing oxygen tensions have beneficial effects in wound repair processes. The antimicrobial properties of oxygen are likely to contribute to a beneficial effect of oxygen therapy through direct bacterial killing [241], especially in the case of infection with anaerobic bacteria [242]. However, it seems that the major beneficial effect comes from the restoration of cellular function, affecting multiple cell types and molecular targets.

In culture, hyperoxia has been reported to promote macrophage chemotaxis [243], phagocytic function in leucocytes [244], fibroblast proliferation and migration [245], and collagen production [246,247]. Earlier studies in our laboratory revealed the reversibility of the adverse effects of hypoxia on myofibroblasts when re-establishing normal oxygen levels.

As oxygen delivery to reparative cells at the wound site relies primarily on diffusion [6], HBOT has been shown to be effective in increasing oxygen tensions in the wound bed [248,249,250,251]. HBOT has been reported to promote wound repair under specific conditions, but the literature is inconclusive [240,252,253,254,255,256]. Exact mechanisms of action of HBOT are still poorly understood, resulting in inconsistent treatment protocols and vague indications [257]. It is likely to have an effect on host defense. Oxygen is the rate-limiting factor in NOX2-mediated ROS production during the respiratory burst. HBOT has been shown to effectively stimulate the respiratory burst and is an accepted adjunct treatment in necrotizing fasciitis and osteomyelitis [258,259].

In chronic wounds, HBOT seems to be especially effective when combined with other treatments, supporting the notion that HBOT might be a potent mechanism for making chronic wounds receptive to targeted treatment [260]. A study on rabbit ear ischemic wounds found that PDGF-BB, a growth factor now widely used in wound therapy, has a much stronger effect when combined with HBOT [254]. The underlying mechanism is likely to be due to the fact that PDGF requires oxygen-derived H_2_O_2_ to function [28].

**Figure 3:**
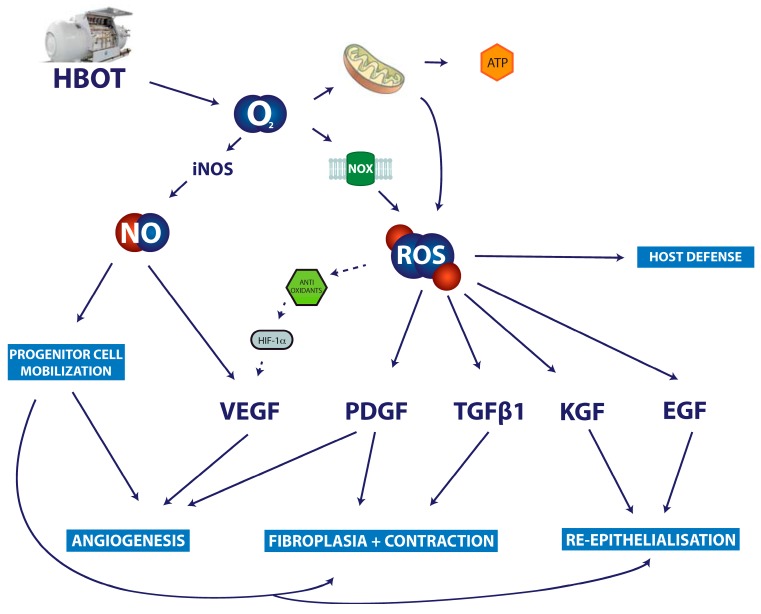
Proposed mechanism of action of hyperbaric oxygen therapy in the treatment of chronic wounds. Solid arrows represent direct promoting interactions and dashed arrows indirect promoting interactions. Besides the increased availability of molecular oxygen leading to enhances energy supply from mitochondria, increased deliberate ROS production by NOX enzymes is likely to enhance wound repair. HBOT has been shown to boost the respiratory burst mediated by NOX2. Other NOX enzymes are likely to play a role in key cytokine signaling pathways improving fibroplasia via PDGF and TGFb1, re-epithelialization via KGF and EGF, and angiogenesis via their implication in HIF1a and PDGF signaling. Furthermore, it has been suggested that HBOT leads to increased progenitor cell mobilization from the bone marrow increasing the availability of nitric oxygen.

HBOT appears to improve angiogenesis during wound repair [261]. In vitro and in vivo, HBOT has been shown to promote secretion of VEGF [262,263]. Results concerning the effects of HBOT on the expression and activity of HIF1 are contradictory. One recent study on rats reported that HBOT improves ischemic wound repair by downregulating HIF-1, which is associated with a reduction of inflammation. [264]. Another study showed increased healing after HBOT, which was associated with increased expression HIF1 and VEGF [263]. The notion that hyperoxia induces HIF1 expression is counterintuitive, but the underlying mechanisms could be found in the increased expression of antioxidants associated with hyperoxia [265]. Oxidative stress induces the expression of the antioxidant thioredoxin, which in return promotes the expression and activity of HIFs [266,267] (see Figure 3). HIF1 is induced in the same way by lactate metabolism [268].

HBOT stimulates keratinocytes, accelerates cornification, and keratinocyte migration [105]. There are several studies suggesting that HBOT improves granulation tissue formation [254,255]. Cultured dermal fibroblasts have been shown to secrete increased levels of TGFβ1 when exposed to HBOT [269]; collagen mRNA production and synthesis in a rat wound repair model were also promoted by HBOT [270]. However, the effects of HBOT on fibroplasia and myofibroblast differentiation during wound repair have not been addressed in detail.

Besides increasing wound oxygenation, other possibilities of generating ROS in the wound bed have been considered for the management of chronic ulcers. Photodynamic therapy (PDT) had first been reported as promising treatment for autoimmune ulcers in 2007 and since then there have been consistent results regarding increased ROS generation by PDT [271,272]. It is currently mainly used in dermato-oncologic settings using the cytotoxic effect of ROS in high concentrations and its applicability in the management of chronic wounds is yet to be established.

## 8. Conclusions

The current literature suggests that oxygen plays a role in all processes of wound repair, and, besides a critical role in oxidative metabolism and bacterial killing, its effects extent to a wide range of redox signaling pathways. The widespread implications of the effects of oxygen during wound repair make HBOT a promising approach for promoting the healing of chronic wounds via tissue oxygenation. Data specifically concerning the role of NOX enzymes in cutaneous wound repair are limited and the enzymes’ function in the tissue-specific response to hypoxia, especially under hyperglycemic conditions, is unknown. Further studies should interrogate the hypothesis that NOX enzymes play a key role in wound repair processes like fibroplasia and in determining the composition of the ECM.
